# Genomic prediction for root and yield traits of barley under a water availability gradient: a case study comparing different spatial adjustments

**DOI:** 10.1186/s13007-023-01121-y

**Published:** 2024-01-12

**Authors:** Biructawit B. Tessema, Miguel A. Raffo, Xiangyu Guo, Simon F. Svane, Lene Krusell, Jens Due Jensen, Anja Karine Ruud, Marta Malinowska, Kristian Thorup-Kristensen, Just Jensen

**Affiliations:** 1 Center for Quantitative Genetics and Genomics, Aarhus University, 8830 Tjele, Denmark; 2 Section of Plant Breeding and Genetics, School of Integrative Plant Sciences, Cornell University, Ithaca, NY USA; 3 Present Address: Danish Pig Research Centre, Danish Agriculture & Food Council, Copenhagen, Denmark; 4 Department of Plant and Environmental Science, University of Copenhagen, 1871 Frederiksberg, Denmark; 5 Sejet Plant Breeding I/S, 8700 Horsens, Denmark; 6 Nordic Seed A/S, 8300 Odder, Denmark; 7 Present Address: Faculty of Biosciences, Department of Plant Science, Norwegian University of Life Sciences (NMBU), Ås, Norway

**Keywords:** Genomic prediction, Spring barley, Semi-field, Roots, Yield, Spatial adjustment

## Abstract

**Background:**

In drought periods, water use efficiency depends on the capacity of roots to extract water from deep soil. A semi-field phenotyping facility (RadiMax) was used to investigate above-ground and root traits in spring barley when grown under a water availability gradient. Above-ground traits included grain yield, grain protein concentration, grain nitrogen removal, and thousand kernel weight. Root traits were obtained through digital images measuring the root length at different depths. Two nearest-neighbor adjustments (M1 and M2) to model spatial variation were used for genetic parameter estimation and genomic prediction (GP). M1 and M2 used (co)variance structures and differed in the distance function to calculate between-neighbor correlations. M2 was the most developed adjustment, as accounted by the Euclidean distance between neighbors.

**Results:**

The estimated heritabilities (${\widehat{h}}^{2}$) ranged from low to medium for root and above-ground traits. The genetic coefficient of variation ($GCV$) ranged from 3.2 to 7.0% for above-ground and 4.7 to 10.4% for root traits, indicating good breeding potential for the measured traits. The highest $GCV$ observed for root traits revealed that significant genetic change in root development can be achieved through selection. We studied the genotype-by-water availability interaction, but no relevant interaction effects were detected. GP was assessed using leave-one-line-out (LOO) cross-validation. The predictive ability (PA) estimated as the correlation between phenotypes corrected by fixed effects and genomic estimated breeding values ranged from 0.33 to 0.49 for above-ground and 0.15 to 0.27 for root traits, and no substantial variance inflation in predicted genetic effects was observed. Significant differences in PA were observed in favor of M2.

**Conclusions:**

The significant $GCV$ and the accurate prediction of breeding values for above-ground and root traits revealed that developing genetically superior barley lines with improved root systems is possible. In addition, we found significant spatial variation in the experiment, highlighting the relevance of correctly accounting for spatial effects in statistical models. In this sense, the proposed nearest-neighbor adjustments are flexible approaches in terms of assumptions that can be useful for semi-field or field experiments.

**Supplementary Information:**

The online version contains supplementary material available at 10.1186/s13007-023-01121-y.

## Introduction

Barley (*Hordeum vulgare* L.) is one of the major cereal crops worldwide. Its production is mainly used for animal feed or malting for alcoholic beverage fabrication (FAO 2016). One of the challenges for barley production is the influence of drought stress [44]. This challenge is exacerbated by the changing climate conditions that pose a great risk to the future water supply for agricultural production [27, 33, 42]. Thus, barley varieties that can overcome periods of drought stress with acceptable productivity are important to ensure future sustainable production. In a water-limited environment, efficient use of water depends on the capacity of roots to extract water from deep soil. In addition, deep roots allow the uptake of nitrogen and other nutrients available in deeper soil layers [27, 33, 42].

Many studies in crop breeding have focused on investigating above-ground traits with a primary focus on grain yield [41, 53, 60], and fewer studies have investigated root traits (Den [14, 24, 25, 31, 35, 51]. The complexity and difficulty of root phenotyping under field conditions are some of the major reasons for the scarcity of root studies. To cope with this issue, a large-scale phenotyping facility to study root growth under semi-field (rain-out shelter) conditions called RadiMax was developed [59]. The RadiMax facility potentially allows testing different plant species in four semi-field shelters of 150 rows of capacity each; see Svane et al. [59] for a full description of the facility. The varieties are assessed under different levels of water availability and genetic differences in root development in crops such as barley can be identified.

Molecular markers have been exploited in plant breeding approaches for the last three decades to improve traits of economic importance. This was initially achieved through marker-assisted selection (MAS, [50],Collard and Mackil, 2007; [10]. The genetic improvements achieved with MAS were mainly relevant for traits affected by the effects of major quantitative trait loci ‘‘QTL’’ [5, 16, 40, 48]. The introduction of genotyping techniques using numerous markers spread over the whole genome has made it possible to perform genomic selection (GS, [39]. GS allows us to capture most QTL effects (major and minor) to predict breeding values for complex traits due to genetic linkage between markers and QTL.

Genomic prediction (GP) uses a biometrical prediction model that is first trained using a population that contains both genotyped and phenotyped individuals. The trained model can then predict genomic breeding values on individuals that have been only genotyped. Such models can also increase the accuracy of predicted breeding values for lines with both phenotypic and genotypic data due to better use of information from genotyped relatives. Genomic estimated breeding values (GEBVs) are calculated as the sum of effects of all dense genetic markers in linkage disequilibrium (LD) with one or more QTLs across the entire genome [23]. GP has been successfully used in barley to predict genomic breeding values for several traits of economic relevance [3, 41, 63] and hybrid performance [45].

Plant experiments are usually affected by spatial variation in the experimental fields that cannot be completely controlled by blocks in the experimental design. For example, intra-block variability can occur due to differences in the availability of nutrients, water and other uncontrolled biotic and abiotic factors [6]. A vast body of scientific literature have been published to address the spatial variation in experimental fields from more than one century ago [2, 12, 21, 43, 56, 61, 68, 69]. Specifying spatial effects in statistical models is important as it can improve the fitting of the model [6, 57].

A classical approach to model spatial variation proposed by Papadakis [43] and developed by Wilkinson et al. [68] is to use the neighbor information to adjust the spatial variation (NNA, nearest neighbor adjustment), which is a particular kind of geostatistical analysis for field trials [47]. Gleeson and Cullis [22] proposed to fit autoregressive-integrated-moving average models (ARIMA) to the plot errors in one direction (rows or columns), which was later extended by Cullis and Gleeson [11] to two directions (rows and columns). Other methods based on spline function have been demonstrated to be efficient in modeling spatial variation in field experiments [46, 65–67]. Detailed reviews of methods for spatial modeling can be found in Martin [38], Hinkelmann and Kempthorne [28] and Piepho et al. [47]. Note that spatial models use information from neighbor observations in order to control environmental variation in the experiments,this needs to be differentiated from the genetic effect of neighbors that, for example, may occur by competition or differential exposure to diseases [8, 36, 37].

In this study, we used a set of spring barley breeding lines provided by breeding companies Nordic Seed A/S and Sejet Plant Breeding, and evaluated under a water availability gradient in the Radimax facility. The water availability gradient was characterized in wet and dry treatments, where above-ground and root traits were collected under each treatment conditions. Details about the facility and treatments are described in the material and methods section. Spatial variation in our experiment was expected along the facility due to differences in soil compaction and other factors. The first motivation for this study came from the need to improve barley lines to withstand drought periods and present higher yields under water-scarce conditions. Our second motivation was to study the effect of different spatial adjustments on the estimation of variance components (VCs), genetic parameters estimation and genomic prediction. The specific objectives of this study were to:

(i) Investigate genetic variation for root development (shallow and deep), grain yield, grain protein content, grain nitrogen removal, and thousand kernel weight in spring barley.
(ii) Investigate genotype-by-treatment (wet and dry) interaction and the potential genetic effects due to neighboring lines in the RadiMax experiment.
(iii) Analyze the possibility of performing genomic prediction for the several root and above-ground traits evaluated in RadiMax using a leave-one-line-out (LOO) cross-validation (CV) strategy.
(iv) To compare two different spatial nearest neighbor adjustments (NNAs) based on (co)variance structures to model spatial relationships between neighboring rows.

## Materials and methods

### Plant materials

A total of 74 Danish spring barley (*Hordeum vulgae* L.) lines provided by Nordic Seed A/S and Sejet plant breeding companies were used in the current experiment (a Principal Component Analysis for genotypes is presented in Additional file 1: Figure S1). The experiment was carried out in 2017 in the RadiMax semi-field root phenotyping facility [59] located at Copenhagen University experimental farm (Latitude 55.66815°N, Longitude 12.30848^o^E). A detailed description of the RadiMax infrastructure can be found in Svane et al. [59].

### RadiMax facility and experiment layout

**Fig. 1:**
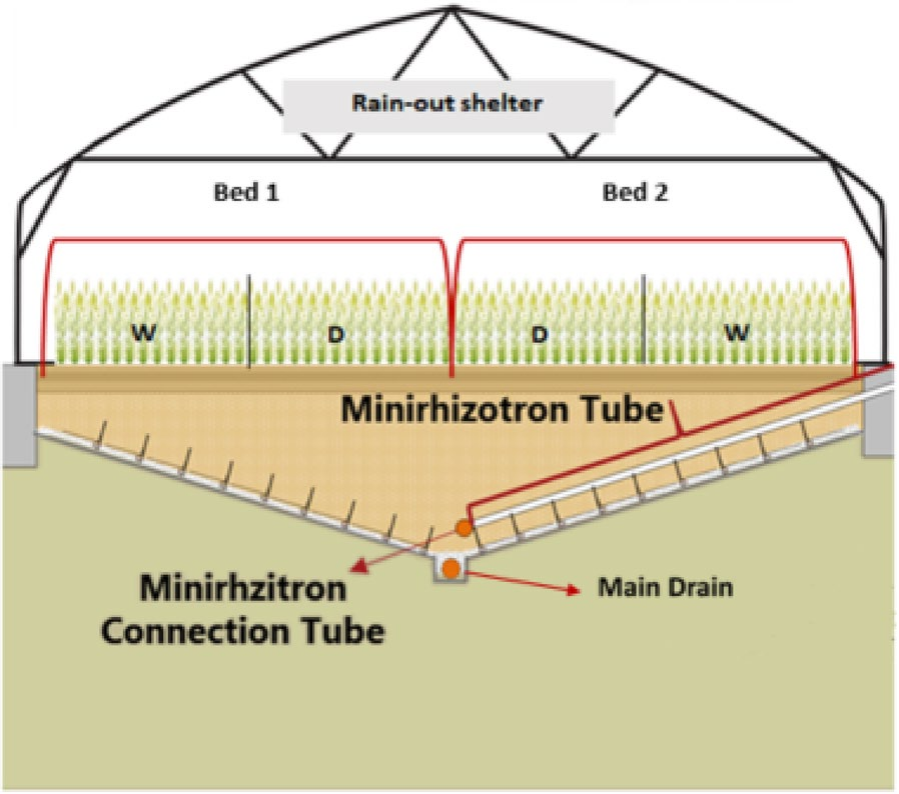
Cross-section of two beds in the semi-field RadiMax facility. W: wet treatment, D: dry treatment. The figure was adapted from Svane et al. [59]

The experiment was established in two beds, each divided into two independent experimental units (half-beds) with 150 rows of length each. The half-beds operated as an independent replicate so that the experiment presented four replicates. As shown in Fig. 1, the RadiMax facility contained concrete walls in the laterals to prevent runoff from adjacent areas and a V-shaped bottom lined with an impermeable plastic membrane. The V-shaped bottom is designed so that the soil depth increases from 1.1 m at the sides to 3.0 m at the deep end of the beds (Fig. 1). A moveable rain-out shelter was used to cover the units during rainy periods. The open ends of the rain-out shelter were covered with a transparent insect net, allowing enough ventilation to reduce warming effects. There were 74 barley lines randomized within each bed. A single barley line was sown in each row with 25 cm row distance. A wet treatment was defined for units one and four, and a dry treatment was defined for units two and three. Treatments are further described in the following heading.

### Treatments

A subsurface irrigation system was used to create a water gradient along the sloped bottom of each experimental bed resulting in different amounts of water available to the plants (Fig. 1). The system uses the principle of capillary movement to distribute water without the assistance of external forces. However, since the distance from soil surface to the water supply was different a dry and wet treatment was defined. The water availability gradient was divided into two parts called treatments (wet and dry) along the rows where the plants were grown (Fig. 1). A row of the experimental bed contains both treatments, where half of the row is wet treatment and the other half is dry treatment. Further details on the RadiMax facility and experimental design can be found in Svane et al. [58].

### Above-ground measures

Above-ground measurements were done separately for wet and dry treatments on half-segment of the rows of each experimental bed. The outermost part of the rows (0.5 m to both sides) were not included for sampling in order to avoid border effects. Four above-ground traits were measured: (1) grain yield (GY) in t/ha, (2) grain protein content (GPC) expressed in percentage, (3) grain nitrogen removal (GNR) in kg/ha and (4) thousand kernel weight (TKW) in grams. GPC was determined using near-infrared spectroscopy (NIR, Intratec grain analyzer, Foss, Hilleroed, Denmark) and expressed as a percentage. GNR was estimated from grain yield and grain protein concentration as: $\frac{GY \times GPC}{6.25}$, where 6.25 is the standard nitrogen-to-protein conversion factor for cereals [30]. TKW was determined as a measure of kernel size using an image-based system for counting the number of seeds in a sample, and it was calculated as the quotient of the sample weight in grams and the total number of seeds, multiplied by 1000.

In total, 1200 observations were recorded for the four above-ground traits (GY, GPC, GNR, TKW). The 1200 observations resulted from collecting samples from beds (2)*half-beds(2)*rows(150)*treatments(2). The following criteria were used for data editing: (1) lines with no observations for any of one trait were removed, (2) data records outside ± 3 standard deviations (SD) from the mean were removed, and (3) lines with no genomic information were removed. After the data cleaning, 1031 observations for 66 lines were available for statistical analysis. Descriptive statistics for above-ground and root traits are presented in Table 1, and a boxplot is included in Additional file 2: Figure S3.

**Table 1:** Descriptive statistics traits

Trait	No. of records	Minimum	Mean	Maximum	SD	CV
GY (t/ha) GPC (%) GNR (kg/ha) TKW (g) TRL (cm) SRL (cm) DRL (cm)	1031 1031 1031 1031 748 771 762	3.52 7.34 51.06 38.39 1.98 0 0	7.07 9.42 106.55 52.83 33.83 19.68 12.85	10.33 11.70 165.16 68.28 108.41 61.40 55.07	1.08 0.76 18.36 4.44 20.96 13.83 10.91	15.28 8.07 17.23 8.41 61.96 70.27 84.90

*GY* grain yield, *GPC* grain protein content, *GNR* grain nitrogen removal, *TKW* thousand kernel weight, *TRL* total root length, *SRL* shallow root length, *DRL* deep root length, *SD* standard deviation, *CV* coefficient of variation (%)

### Root images

Root images were taken in minirhizotrons (MR), transparent tubes installed 40 cm above the sloping bottom for half of the experimental beds (Fig. 1). Tubes were guided through holes in the concrete wall to facilitate capturing of root images in the range across a soil depth of 0.4 to 2.7 m. The MR tube has 70 mm outer diameter, 60 mm inner diameter and a total length of 5.5 m. The MR tubes were placed 25 cm apart. In total, there were 300 MR tubes, with 150 MR tubes for each MR-equipped bed. A root image (19.8 cm.^2^) facing upward was taken for every 5 cm within the tube, using a custom-built multispectral MR imaging system. The system offers automated quantification of the length of living root structures, by a multivariate grouping of pixels based on differences in reflectance and suppression of background noise by the use of a vesselness enhancement filter algorithm. For a full description of the image acquisition system and the training procedure of the image analysis procedure see Svane et al. [58]

Root images were taken at three different time points during the growth season. Time points 1, 2, and 3 were on June 13, June 28, and July 19 of year 2017, respectively. The number of images for time points 1, 2, and 3 were 9,253, 13,485, and 12,106, respectively. The following criteria were used for editing the image data: (1) images with no observations were removed, (2) data records outside ± 3 SD from the mean were removed, and (3) lines with no genomic information were removed. After data cleaning, 6027, 10,032, and 11,642 images were available for analysis for time points 1, 2, and 3, respectively. On average, 21, 35, and 41 images were available per line for time points 1, 2 and 3, respectively. For the genetic analysis, the root data from the three time points were used as repeated observations in the model, resulting in a potential number of 900 observations from beds(2)*Minirhizotrons(150)*time-points(3). The reason for combining the data as repeated observations was that the initial analysis for each time point separately showed relatively homogeneous genetic variance across time points, and the combined analysis tended to capture higher genetic variance.

For the root data analysis, we have summarized the root data for each minirhizotron and time-point in three different ways:

1. Total root length (TRL) calculated by summing the visible root length present in each image for each MR tube.
2. Shallow root length (SRL) as the sum of visible root length in the interval of 100–120 cm of soil depth.
3. Deep root length (DRL) as the sum of visible root length in the interval of 120–180 cm of soil depth.

### Genomic data

Samples consisting of seven flag leaves per line taken from wet treatment were used for transcriptome sequencing (RNAseq). RNA was extracted from leaf tissues using the Total Plant RNA kit (Sigma-Aldrich, Schnelldorf, Germany). Gene expression data was generated via RNA-seq on the Illumina HiSeq4000 platform (2 × 100 bp, ~ 20 M reads per sample) by the Beijing Genomics Institute (BGI, Shenzhen, China). The initial data set included 135,329 SNPs and the following SNP filtering criteria was applied: (1) SNPs and lines with more than 10% missing data were excluded, (2) SNPs with minor allele frequency (MAF) lower than 3% were excluded. After filtering, 60,048 SNPs were available for the analysis.

The SNP data set was then used to compute the genomic relationship matrix $({\varvec{G}})$ following the first method of VanRaden [64] as follows:

$$G = \frac{{MM^{\prime}}}{{2\Sigma P_{j} (1 - p_{j} )}}$$

where *M* is the centered genotypic matrix, and ${p}_{j}$ is the minor allele frequency of *j*^*th*^ SNP. A Heatmap of ${\varvec{G}}$ is presented in Additional file 1: Figure S2.

### Statistical models

Initially, several models were tested to estimate VCs for above-ground and root traits. Investigation of spatial effect separately for areas under wet and dry treatments for above-ground trait, and by time-point for root traits, revealed heterogenous spatial variances. Thus, we considered heterogeneous spatial variance for each treatment (dry and wet) for above ground traits, and for time points 1, 2, and 3 for root traits. Two approaches based on covariance structures with different distance functions to compute neighbor relationships were used (Additional file 3: Figure S4). The two modeling approaches were referred to as M1 and M2, for the above-ground models as AM1 and AM2 and the root models as RM1 and RM2.

#### *Above-ground models (AM*)

The AM1 model was defined as follows:

AM1 $${\varvec{y}}=\boldsymbol{ }{\varvec{X}}{\varvec{b}}+\boldsymbol{ }{{\varvec{Z}}}_{{\varvec{g}}}{\varvec{g}}+\boldsymbol{ }{{\varvec{Z}}}_{{\varvec{l}}}{\varvec{l}}+\boldsymbol{ }{{\varvec{Z}}}_{{\varvec{n}}{\varvec{g}}}{{\varvec{n}}}_{{\varvec{g}}}+{{\varvec{Z}}}_{{\varvec{n}}{\varvec{l}}}{{\varvec{n}}}_{{\varvec{l}}}+{{\varvec{Z}}}_{{\varvec{r}}}{\varvec{r}}+{{\varvec{Z}}}_{{\varvec{t}}{\varvec{g}}}{{\varvec{t}}}_{{\varvec{g}}}+\boldsymbol{ }{{\varvec{Z}}}_{{\varvec{t}}{\varvec{l}}}{{\varvec{t}}}_{{\varvec{l}}}+\boldsymbol{ }{{\varvec{Z}}}_{{\varvec{s}}1}{{\varvec{s}}}_{1}+\boldsymbol{ }{{\varvec{Z}}}_{{\varvec{s}}2}{{\varvec{s}}}_{2}+\boldsymbol{ }{\varvec{e}}$$

where ${\varvec{y}}$ is the vector of phenotypes for GY, GPC, GNR, or TKW; ${\varvec{X}}$ is the design matrix for the fixed effects; ${\varvec{b}}$ is the vector of fixed effects (unit-bed-treatment); ${\varvec{g}}$ is the vector of additive genomic breeding values of the lines with ${\varvec{g}} \sim N(0,{\varvec{G}}{\sigma }_{g}^{2})$, where ${\varvec{G}}$ is the genomic relationship matrix and ${\sigma }_{g}^{2}$ the additive genomic variance; ${\varvec{l}}$ is the vector of line effects that include non-additive genetic effects and potential additive effects not explained by the genomic information with ${\varvec{l}} \sim N(0,{\varvec{I}}{\sigma }_{l}^{2})$, where ${\sigma }_{l}^{2}$ is the variance of line effects; ${{\varvec{n}}}_{g}$ is the vector of additive genomic effects due to neighboring effects with ${{\varvec{n}}}_{{\varvec{g}}} \sim N(0,{\varvec{G}}{\sigma }_{ng}^{2})$, with ${\varvec{G}}$ as previously defined and ${\sigma }_{ng}^{2}$ the genomic additive variance due to neighboring effects; ${{\varvec{n}}}_{l}$ is the vector of neighbor line effects due to neighboring effects including non-additive effects and potential additive effects not explained by the genomic information with ${{\varvec{n}}}_{{\varvec{l}}} \sim N(0,{\varvec{I}}{\sigma }_{nl}^{2})$, where ${\sigma }_{nl}^{2}$ is the variance of neighbor line effects; ${\varvec{r}}$ is the vector for row effects with ${\varvec{r}}\boldsymbol{ }\sim \boldsymbol{ }N(0, {\varvec{I}}{\sigma }_{r}^{2})$, where ${\sigma }_{r}^{2}$ is the row variance, ${{\varvec{t}}}_{g}$ and ${{\varvec{t}}}_{l}$ are vectors of genotype by treatment interactions, ${{\varvec{t}}}_{{\varvec{g}}}$ with covariance structure $${{\varvec{t}}}_{g} \sim N(0,\left[\begin{array}{cc}G& 0\\ 0& G\end{array}\right]{\sigma }_{tg}^{2})$$ and ${{\varvec{t}}}_{l} \sim N(0, {\varvec{I}}{\sigma }_{tl}^{2})$, where ${\sigma }_{tg}^{2}$ and ${\sigma }_{tl}^{2}$ are the genomic additive-by-treatment interaction variance and the line-by-treatment interaction variance, respectively; ${{\varvec{s}}}_{1}$ and ${{\varvec{s}}}_{2}$ are the vectors of spatial effects for wet and dry treatments, respectively, with ${{\varvec{s}}}_{1} \sim N(0,{{\varvec{S}}}_{knn}{\sigma }_{s1}^{2})$ and ${{\varvec{s}}}_{2} \sim N(0,{{\varvec{S}}}_{knn}{\sigma }_{s2}^{2})$, where ${{\varvec{S}}}_{knn}$ is a spatial correlation matrix [49] and ${\sigma }_{s1}^{2}$ and ${\sigma }_{s2}^{2}$ is the variance of ${{\varvec{s}}}_{1}$ and ${{\varvec{s}}}_{2}$ effects; ${\varvec{e}}$ is a vector of random residual effect with ${\varvec{e}} \sim N(0,{\varvec{I}}{\sigma }_{e}^{2})$, where ${\sigma }_{e}^{2}$ is the residual variance. The spatial effects were defined as the combination of 11 spatial sub-components integrated by the row centered in the row of the observation (i.e. target row) and the ten neighboring rows (five to the left and five to the right). Virtual rows were added to complete empty row spaces [62]. The ${{\varvec{S}}}_{knn}$ matrix was computed as:

$$S_{kkn} = \frac{{X_{1} X_{1}^{\prime } }}{{{{tr(X_{1} X_{1}^{\prime } )} \mathord{\left/ {\vphantom {{tr(X_{1} X_{1}^{\prime } )} n}} \right. \kern-0pt} n}}}$$

where ${\mathbf{X}}_{1}$ is an n × q matrix, with n = number of observations (i.e., number of real rows), and q = number of real plus virtual rows. The ${{\mathbf{X}}_{1}}_{{\text{n}}\times {\text{q}}}$ is an indicator matrix relating observations to spatial effects in ${{\varvec{S}}}_{knn}$, $tr$ is the trace (sum of diagonal elements) and $n$ the total number of rows.

The AM2 model had the same fixed and random effects as AM1, with the same definition for${\varvec{y}}$,${\varvec{X}}$,${{\varvec{Z}}}_{{\varvec{n}}}$,${\varvec{b}}$,${\varvec{g}}$,${\varvec{l}}$,${{\varvec{n}}}_{{\varvec{g}}}$,${{\varvec{n}}}_{{\varvec{l}}}$,${\varvec{r}}$,${{\varvec{t}}}_{{\varvec{g}}}$,${{\varvec{t}}}_{{\varvec{l}}}$, and${\varvec{e}}$, but a different definition for ${{\varvec{s}}}_{1}$ and${{\varvec{s}}}_{2}$, which accounted for the Euclidean distance among neighbors. The spatial effects in AM2 were defined as ${{\varvec{s}}}_{1} \sim N(0,{{\varvec{S}}}_{euc}{\sigma }_{s1}^{2})$ and${{\varvec{s}}}_{2} \sim N(0,{{\varvec{S}}}_{euc}{\sigma }_{s2}^{2})$, where ${{\varvec{S}}}_{euc}$ is a spatial correlation matrix and ${\sigma }_{s1}^{2}$ and ${\sigma }_{s2}^{2}$ is the variance of ${{\varvec{s}}}_{1}$ and ${{\varvec{s}}}_{2}$ effects. The ${{\varvec{S}}}_{euc}$ matrix was computed as${{\varvec{S}}}_{euc}=\frac{{\mathbf{X}}_{2}{{\mathbf{X}}_{2}}^{\mathbf{^{\prime}}}}{tr\left({\mathbf{X}}_{2}{{\mathbf{X}}_{2}}^{\mathbf{^{\prime}}}\right)/n}$, where ${\mathbf{X}}_{2}$ is an n × q matrix, with n = number of observations and q = number of real plus virtual rows. The ${{\mathbf{X}}_{2}}_{{\text{n}}\times {\text{q}}}$ matrix was built first relating the target rows with the neighbors within a distance ≤ 2.75 m (equivalent to the distance of 11 rows as defined for the${{\varvec{S}}}_{knn}$), and second by scaling the Euclidean distance between the target rows and their neighbors (${d}_{ij}$) by the maximum distance ${d}_{max}$ = 2.75 m [13, 15]. Virtual rows were added to complete empty row spaces. This approach was implemented using the "adespatial" R package [15]. Note that the spatial effect in M1 and M2 are both based on nearest neighbor adjustment (NNA) but differ in the distance function used to compute the relationship between neighbors $.$ A scatter plot of the between neighbor correlations in ${{\varvec{S}}}_{knn}$ and ${{\varvec{S}}}_{euc}$ as a function of neighbors distance, and a heatmap of the ${{\varvec{S}}}_{knn}$ matrix is provided in Additional file 3. Note that the main difference between ${{\varvec{S}}}_{knn}$ and ${{\varvec{S}}}_{euc}$ are that ${{\varvec{S}}}_{euc}$ weight higher correlations for closer neighbors and lower for more distant neighbors compared to${{\varvec{S}}}_{knn}$.

#### *Root models (RM*)

Two models, RM1 and RM2, were developed for root traits. Unlike the AM models, the RM models did not include neighboring line effects because of the high model complexity. The RM1 model was defined as follows:

RM1 $${\varvec{y}}=\boldsymbol{ }{\varvec{X}}{\varvec{b}}+{{\varvec{Z}}}_{{\varvec{g}}}{\varvec{g}}+\boldsymbol{ }{{\varvec{Z}}}_{{\varvec{l}}}{\varvec{l}}+\boldsymbol{ }{{\varvec{Z}}}_{{\varvec{r}}}{\varvec{r}}+{{\varvec{Z}}}_{{\varvec{s}}1}{{\varvec{s}}}_{1}+\boldsymbol{ }{{\varvec{Z}}}_{{\varvec{s}}2}{{\varvec{s}}}_{2}+{{\varvec{Z}}}_{{\varvec{s}}3}{{\varvec{s}}}_{3}+\boldsymbol{ }{\varvec{e}}$$

where ${\varvec{y}}$ is the vector of phenotypes for TRL, SRL, and DRL; ${\varvec{X}}$ is the design matrix for the fixed factors; ${\varvec{b}}$ is the vector of fixed effects (bed-camera-time); and ${{\varvec{Z}}}_{{\varvec{n}}}$, ${\varvec{g}}$, ${\varvec{l}}$, ${\varvec{r}}$, and ${\varvec{e}}$ were defined as in AM1; ${{\varvec{s}}}_{1}$, ${{\varvec{s}}}_{2}$, and ${{\varvec{s}}}_{3}$ are spatial variances for time-point 1, 2, and 3, respectively, with ${{\varvec{s}}}_{1} \sim N(0,{{\varvec{S}}}_{knn}{\sigma }_{s1}^{2})$, ${{\varvec{s}}}_{2} \sim N(0,{{\varvec{S}}}_{knn}{\sigma }_{s2}^{2})$, and ${{\varvec{s}}}_{3} \sim N(0,{{\varvec{S}}}_{knn}{\sigma }_{s3}^{2})$. The RM2 model had the same fixed and random effects as RM1, but with ${{\varvec{s}}}_{1}$, ${{\varvec{s}}}_{2}$, and ${{\varvec{s}}}_{3}$ using the ${{\varvec{S}}}_{euc}$ covariance structure instead of ${{\varvec{S}}}_{knn}$.

### Variance components and genetic parameters

Variance components were estimated by Restricted Maximum Likelihood (REML) using the Average Information (AI-REML) module in DMU software [34]. The narrow (${\widehat{h}}^{2}$) and broad-sense (${\widehat{H}}^{2}$) heritabilities were estimated at the level of half-rows for the different treatments for AM and different time-points for RM models. The narrow (${h}^{2}$) and broad-sense heritabilities (${H}^{2}$) were estimated for all models as ${\widehat{h}}^{2}=d(\mathbf{G}){ \widehat{\sigma }}_{g}^{2}/{ \widehat{\sigma }}_{P}^{2}$ and ${\widehat{H}}^{2}=({\widehat{\sigma }}_{l}^{2}+d(\mathbf{G}){ \widehat{\sigma }}_{g}^{2})/{ \widehat{\sigma }}_{P}^{2}$, where $d(\mathbf{G})$ is the average of diagonal elements of the genomic relationship matrix $d\left(\mathbf{G}\right)$ = 1.856, ${\widehat{\sigma }}_{g}^{2}$ is the estimated additive genomic variance,${\widehat{\sigma }}_{l}^{2}$ is the estimated variance of line effects, and ${\widehat{\sigma }}_{P}^{2}$ is the estimated half-row phenotypic variance. The ${\widehat{\sigma }}_{P}^{2}$ for AM models was calculated as:

AM $${\widehat{\sigma }}_{{P}_{i}}^{2}= {d(\mathbf{G})\widehat{\sigma }}_{g}^{2} + {\widehat{\sigma }}_{l}^{2}+ {2d(\mathbf{G})\widehat{\sigma }}_{gn}^{2}+ {2\widehat{\sigma }}_{ln}^{2}+{\widehat{\sigma }}_{r}^{2}+d(\mathbf{G}){\widehat{\sigma }}_{tg}^{2}+{\widehat{\sigma }}_{tl}^{2}+{\widehat{\sigma }}_{{s}_{i}}^{2}+{\widehat{\sigma }}_{e}^{2}$$

where $i$ is 1 or 2 for wet and dry treatments, respectively; the “hat” denotes the estimate of the parameter for ${\sigma }_{g}^{2}$, ${\sigma }_{l}^{2}$, ${\sigma }_{ng}^{2}$, ${\sigma }_{nl}^{2}$, ${\sigma }_{r}^{2}$, ${\sigma }_{tg}^{2}$, ${\sigma }_{tl}^{2}$, ${\sigma }_{{s}_{i}}^{2}$ ($i=1$ and $2$ for wet and dry treatment, respectively), and ${\sigma }_{e}^{2}$. The ${\widehat{\sigma }}_{P}^{2}$ for RM models was calculated as:

$${\widehat{\sigma }}_{{P}_{k}}^{2}={d(\mathbf{G})\widehat{\sigma }}_{g}^{2} + {\widehat{\sigma }}_{l}^{2}+ {\widehat{\sigma }}_{r}^{2}+{\widehat{\sigma }}_{{s}_{k}}^{2}+{\widehat{\sigma }}_{e}^{2}$$

where $k$ is 1, 2, or 3 for time-point 1, 2, and 3, respectively, ${\widehat{\sigma }}_{g}^{2}$, ${\widehat{\sigma }}_{l}^{2}$, ${\widehat{\sigma }}_{r}^{2}$, and ${\widehat{\sigma }}_{e}^{2}$ were defined as for the AM models, and ${\widehat{\sigma }}_{{s}_{k}}^{2}$ is the estimated variance for the spatial effect $k$ ($k=1$, 2, and $3$ for time-point 1, 2, and 3, respectively).

The genetic coefficient of variation ($GCV$) was calculated for all above-ground and root traits as $GCV= \frac{{\sigma }_{g}}{\overline{x} }*100$, where ${\sigma }_{g}$ is the square root of the additive genomic variance and $\overline{x }$ the general average for each trait.

### Genomic predictions

Models were validated using a leave-one-line-out (LOO) cross-validation (CV) strategy, where the GEBV of each line was predicted from a model trained on all the other lines. This validation strategy first estimates variance components and fixed effects from the full data set. Then estimates of the fixed effects were subtracted from the phenotype to get corrected phenotype $({y}_{c})$. For breeding value prediction, one line was left out at a time and prediction for the left-out line was done based on the rest of the lines. This process continued until all lines were predicted. The predictive ability (PA) of models were determined as the correlation between the phenotypic values corrected for the fixed effects and GEBVs. The maximum potential PA was computed as $\sqrt{n{h}^{2}/(1+\left(n-1\right){h}^{2})}$ [9, 19], where ${\text{n}}$ is the average number of line repetitions (${\text{n}}=$15.9 for above-ground traits and ${\text{n}}=$11.5 for root traits including the three time points). Note that the prediction accuracy (ACC) can be estimated by scaling the PA by the estimated maximum potential PA. The regression coefficient of predicted genetic values obtained with whole phenotypic information on predicted values obtained with partial phenotypic information was used as an estimate for variance inflation: ${{\varvec{b}}}_{w,p}= \frac{cov({\widehat{g}}_{w}, {\widehat{g}}_{p})}{var({\widehat{g}}_{p})}$ [32]. An ordinary non-parametric bootstrapping with replacement, full sample size, and 10,000 replication was used to obtain standard errors for PA and ${{\varvec{b}}}_{w,p}$. The PA from the different model was assessed using a Hotelling-Wiliams t-test [17]. Differences were considered significant for a *P*-value lower than 0.01.

## Results

### Treatment estimates and variance component estimates for above-ground traits

The main treatment effect (wet and dry) was estimated as fixed effects in AM1 and AM2. Similar treatment estimates were observed between models. The between-model average estimates for wet treatment were 7.20, 9.30, 107.02, and 53.42 for GY, GPC, GNR and TKW, respectively, and 6.91, 9.59, 106.72, and 52.40 for GY, GPC, GNR and TKW for dry treatment, respectively.

The VCs and genetic parameters estimates for above-ground traits are presented in Table 2. The AM1 had an estimated additive genomic variance (${\widehat{\sigma }}_{g}^{2}$) of 0.23, 0.09, 50.1, and 8.97 for GY, GPC, GNR and TKW, respectively, and it was higher than the estimated line variance (${\widehat{\sigma }}_{l}^{2}$) for all above-ground traits. The estimated row variance (${\widehat{\sigma }}_{r}^{2}$) was 0.13, 0.03, 34.3, and 0.55 for GY, GPC, GNR and TKW, respectively. The estimated genetic variance of effect of neighbors (${\widehat{\sigma }}_{{n}_{g}}^{2}$ and ${\widehat{\sigma }}_{{n}_{l}}^{2}$) and genetic-by-treatment interaction (${\widehat{\sigma }}_{tg}^{2}$ and ${\widehat{\sigma }}_{tl}^{2}$) was low for all traits. The spatial variances estimated with AM1 for the wet treatment (${\widehat{\sigma }}_{s1}^{2}$) were 0.12, 0.26, 89.6, and 0.40 for GY, GPC, GNR and TKW, respectively, which were higher than for the dry treatment and represented 11.2, 59.2, 29.4, and 2.6% of the estimated phenotypic variance (${\widehat{\sigma }}_{P}^{2}$). The ${\widehat{\sigma }}_{s2}^{2}$ for dry treatment were 0.06, 0.16, 43.9, and 1.41, representing 5.9, 47.7, 16.9, and 7.8% for GY, GPC, GNR and TKW, respectively.

**Table 2:** Variance components and genetic parameter estimates for above-ground traits

Trait	${\widehat{\sigma }}_{g}^{2}$	${\widehat{\sigma }}_{l}^{2}$	${\widehat{\sigma }}_{{n}_{g}}^{2}$	${\widehat{\sigma }}_{{n}_{l}}^{2}$	${\widehat{\sigma }}_{r}^{2}$	${\widehat{\sigma }}_{tg}^{2}$	${\widehat{\sigma }}_{tl}^{2}$	${\widehat{\sigma }}_{s1}^{2}$	${\widehat{\sigma }}_{s2}^{2}$	${\widehat{\sigma }}_{e}^{2}$	${\widehat{h}}^{2}$^a^	${\widehat{H}}^{2}$^a^	$GCV$(%)
GY_AM1	0.23	0.11	3.7E−07	2.5E−07	0.13	0.01	0.01	0.12	0.06	0.48	0.22	0.32	6.8
GY_AM2	0.24	0.09	9.9E−04	2.6E−07	0.14	0.01	0.01	0.15	0.04	0.50	0.22	0.31	7.0
GPC_AM1	0.09	0.01	2.6E−08	1.4E−07	0.03	3.5E-03	1.8E−03	0.26	0.16	0.04	0.24	0.27	3.2
GPC_AM2	0.10	0.01	3.1E−08	1.0E−07	0.10	2.6E-03	4.3E−03	0.45	0.18	0.05	0.19	0.20	3.4
GNR_AM1	50.1	12.6	1.1E−06	0.65	34.3	3.9E-06	5.32	89.6	43.9	112.2	0.18	0.23	6.6
GNR_AM2	55.7	7.24	0.20	1.24	48.8	0.02	8.27	111.8	40.4	118.7	0.18	0.20	7.0
TKW_AM1	8.97	4.49	9.7E−03	6.0E−08	0.55	0.01	3.0E−10	0.44	1.41	2.62	0.52	0.78	5.7
TKW_AM2	8.66	4.79	5.2E−10	2.0E−10	0.67	0.01	7.3E−10	0.20	1.23	3.02	0.49	0.76	5.6

*GY* grain yield, *GPC* grain protein content, *GNR* grain nitrogen removal, *TKW* thousand kernel weight, ${\widehat{{\varvec{\sigma}}}}_{{\varvec{g}}}^{2}$ estimated additive genomic variance, ${\widehat{{\varvec{\sigma}}}}_{{\varvec{l}}}^{2}$: estimated line variance, ${\widehat{{\varvec{\sigma}}}}_{{{\varvec{n}}}_{{\varvec{g}}}}^{2}$: estimated additive genomic variance for the neighbor lines, ${\widehat{{\varvec{\sigma}}}}_{{{\varvec{n}}}_{{\varvec{l}}}}^{2}$: estimated line variance for the neighbor lines, ${\widehat{{\varvec{\sigma}}}}_{{\varvec{r}}}^{2}$: estimated row variance, ${\widehat{{\varvec{\sigma}}}}_{{\varvec{t}}{\varvec{g}}}^{2}$: estimated variance for genomic additive-by-treatment interaction, ${\widehat{{\varvec{\sigma}}}}_{{\varvec{t}}{\varvec{l}}}^{2}$: estimated variance for line-by-treatment interaction, ${\widehat{{\varvec{\sigma}}}}_{{\varvec{s}}1}^{2}$: estimated spatial variance for wet treatment, ${\widehat{{\varvec{\sigma}}}}_{{\varvec{s}}2}^{2}$: estimated spatial variance for dry treatment, ${\widehat{{\varvec{\sigma}}}}_{{\varvec{e}}}^{2}$: estimated residual variance, ${\widehat{{\varvec{h}}}}^{2}$: narrow-sense heritability, ${\widehat{{\varvec{H}}}}^{2}$: broad-sense heritability, ${\varvec{G}}{\varvec{C}}{\varvec{V}}$: genetic coefficient of variation.

^a^Heritabilities are presented as an average over treatment. Standard errors of variance estimates are presented in Additional file 5

The AM2 model presented similar VCs estimates to AM1 for ${\widehat{\sigma }}_{g}^{2}$, ${\widehat{\sigma }}_{l}^{2}$, ${\widehat{\sigma }}_{{n}_{g}}^{2}$, ${\widehat{\sigma }}_{{n}_{l}}^{2}$, ${\widehat{\sigma }}_{tg}^{2}$ and ${\widehat{\sigma }}_{tl}^{2}$ for all above-ground traits. The estimated spatial variances with AM2 for the wet treatment (${\widehat{\sigma }}_{s1}^{2}$) were 0.15, 0.45, 111.6, and 0.20, representing 13.3, 63.3, 31.7, and 1.1% for GY, GPC, GNR and TKW, respectively, and for the dry treatment (${\widehat{\sigma }}_{s2}^{2}$) were 0.04, 0.18, 40.4, and 1.23, representing 4.2, 40.8, 14.3, and 6.7% for GY, GPC, GNR and TKW, respectively. The estimated row (${\widehat{\sigma }}_{r}^{2}$), spatial (${\widehat{\sigma }}_{s1}^{2}$ and ${\widehat{\sigma }}_{s2}^{2}$), and residuals (${\widehat{\sigma }}_{e}^{2}$) variances with AM2 were, in general, larger than for AM1. Note that there were three model effects, row (${\varvec{r}}$), spatial (${\varvec{s}}$), and residual (${\varvec{e}}$), describing the environmental variance in AM1 and AM2.

Analysis of residuals was performed for above-ground traits (Additional file 4: Figure S6). Normal distribution and homoscedasticity of residual variances were observed for all traits.

### Genetic parameters for above-ground traits

The narrow (${\widehat{h}}^{2}$) and broad sense heritabilities (${\widehat{H}}^{2}$) at half-row level were estimated for AM1 and AM2 (average values for the treatment are reported in Table 2). The ${\widehat{h}}^{2}$ and ${\widehat{H}}^{2}$ for AM1 and AM2 varied for the different traits, with higher values reported for TKW (${\widehat{h}}^{2}$ = 0.49 to 0.52 and ${\widehat{H}}^{2}$ = 0.76 to 0.78), lower for GNR (${\widehat{h}}^{2}$ = 0.18 and ${\widehat{H}}^{2}$ = 0.20 to 0.23), and intermediate values for GY (${\widehat{h}}^{2}$ = 0.22 and ${\widehat{H}}^{2}$ = 0.31 to 0.32) and GPC (${\widehat{h}}^{2}$ = 0.19 to 0.24 and ${\widehat{H}}^{2}$ = 0.20 to 0.27). In general, ${\widehat{h}}^{2}$ and ${\widehat{H}}^{2}$ were similar for the wet and dry treatments, but some small differences were observed depending on the traits analyzed, with a trend of slightly higher heritability for the dry treatment. AM1 and AM2 presented similar trends on ${\widehat{h}}^{2}$ and ${H}^{2}$ for GY, GNR and TKW; however, the AM2 revealed higher heritabilities than AM1 for GPC.

The genetic coefficient of variation ($GCV$) was estimated for AM1 and AM2 (Table 2), and similar $GCV$ were observed for both models. The AM1 had a $GCV$ of 6.8, 3.2, 6.6, and 5.7 for GY, GPC, GNR and TKW, respectively, and the AM2 presented a $GCV$ of 7.0, 3.4, 7.0, and 5.6 for GY, GPC, GNR and TKW, respectively.

### Variance component estimates for root traits

The VCs and genetic parameter estimates for root traits are presented in Table 3. The RM1 had a ${\widehat{\sigma }}_{g}^{2}$ of 10.13, 4.02, and 0.69 for TRL, SRL and DRL, respectively. The ${\widehat{\sigma }}_{g}^{2}$ captured most of the genetic variation in the RM1 and ${\widehat{\sigma }}_{l}^{2}$ were close to zero for all traits. The estimated row variance (${\widehat{\sigma }}_{r}^{2}$), spatial variance for time-point 1 (${\widehat{\sigma }}_{s1}^{2}$), 2 (${\widehat{\sigma }}_{s2}^{2}$), and 3 (${\widehat{\sigma }}_{s3}^{2}$) captured most of the variation for all root traits. The ${\widehat{\sigma }}_{r}^{2}$ were 127.82, 109.82, and 34.46 for TRL, SRL and DRL, respectively. The ${\widehat{\sigma }}_{s1}^{2}$ for RM1 were 61.89, 16.76, and 6.30, and represented 24.7, 11.2, and 10.4% of ${\widehat{\sigma }}_{P}^{2}$ for TRL, SRL and DRL, respectively; ${\widehat{\sigma }}_{s2}^{2}$ were 88.45, 1.93, and 29.88 (32.0, 1.4, and 35.6%, respectively) and ${\widehat{\sigma }}_{s3}^{2}$ were 232.72, 26.42, and 67.70 (55.3, 16.6, and 55.5%, respectively) for TRL, SRL and DRL, respectively.

**Table 3:** Variance components and genetic parameter estimates for root traits

Trait	${\widehat{\sigma }}_{g}^{2}$	${\widehat{\sigma }}_{l}^{2}$	${\widehat{\sigma }}_{r}^{2}$	${\widehat{\sigma }}_{s1}^{2}$	${\widehat{\sigma }}_{s2}^{2}$	${\widehat{\sigma }}_{s3}^{2}$	${\widehat{\sigma }}_{e}^{2}$	Time 1		Time 2		Time 3		$GCV$ (%)
								${\widehat{h}}^{2}$	${\widehat{H}}^{2}$	${\widehat{h}}^{2}$	${\widehat{H}}^{2}$	${\widehat{h}}^{2}$	${\widehat{H}}^{2}$	
TRL_RM1	10.13	2.6E−07	127.82	61.89	88.45	232.72	50.00	0.04	0.04	0.04	0.04	0.02	0.02	9.4
TRL_RM2	8.86	9.5E−07	193.46	95.16	53.37	238.64	60.82	0.03	0.03	0.03	0.03	0.02	0.02	8.8
SRL_RM1	4.02	6.5E−07	109.82	16.76	1.93	26.42	19.00	0.03	0.03	0.03	0.03	0.03	0.03	10.2
SRL_RM2	4.23	1.1E−07	110.64	29.55	2.15	66.19	19.67	0.03	0.03	0.03	0.03	0.02	0.02	10.4
DRL_RM1	0.69	4.2E−08	34.46	6.30	29.88	67.70	19.06	0.01	0.01	0.01	0.01	0.01	0.01	6.5
DRL_RM2	0.36	8.6E−08	40.25	1.70	44.11	159.85	24.07	0.005	0.005	0.003	0.003	0.002	0.002	4.7

*TRL* total root length, *SRL* shallow root length, *DRL* deep root length, ${\widehat{{\varvec{\sigma}}}}_{{\varvec{g}}}^{2}$: estimated additive genomic variance, ${\widehat{{\varvec{\sigma}}}}_{{\varvec{l}}}^{2}$: estimated line variance, ${\widehat{{\varvec{\sigma}}}}_{{\varvec{r}}}^{2}$: estimated row variance, ${\widehat{{\varvec{\sigma}}}}_{{\varvec{s}}1}^{2}$: estimated spatial variance for time-point 1, ${\widehat{{\varvec{\sigma}}}}_{{\varvec{s}}2}^{2}$: estimated spatial variance for time-point 2, ${\widehat{{\varvec{\sigma}}}}_{{\varvec{s}}3}^{2}$: estimated spatial variance for time-point 3, ${\widehat{{\varvec{\sigma}}}}_{{\varvec{e}}}^{2}$: estimated residual variance, ${{\varvec{h}}}^{2}$: narrow-sense heritability, ${{\varvec{H}}}^{2}$: broad-sense heritability, ${\varvec{G}}{\varvec{C}}{\varvec{V}}$: genetic coefficient of variation. Standard errors of variance estimates are presented in Additional file 5

The RM2 had similar ${\widehat{\sigma }}_{g}^{2}$ to RM1 for TRL and SRL, but had lower ${\widehat{\sigma }}_{g}^{2}$ (${\widehat{\sigma }}_{g}^{2}$ = 0.36) than RM1 for DRL. The ${\widehat{\sigma }}_{l}^{2}$ for RM2 was close to zero for all root traits. Similarly to RM1, in RM2 the ${\widehat{\sigma }}_{r}^{2}$, ${\widehat{\sigma }}_{s1}^{2}$, ${\widehat{\sigma }}_{s2}^{2}$, and ${\widehat{\sigma }}_{s3}^{2}$ captured most of the variation for all root traits. The ${\widehat{\sigma }}_{r}^{2}$ were 193.46, 110.64, and 40.25 for TRL, SRL and DRL, respectively. The ${\widehat{\sigma }}_{s1}^{2}$ for RM1 were 95.16, 29.55, and 1.70, and represented 26.6, 18.0, and 2.6% of ${\widehat{\sigma }}_{P}^{2}$ for TRL, SRL and DRL, respectively; ${\widehat{\sigma }}_{s2}^{2}$ were 53.37, 2.15, and 44.11 (16.9, 1.6, and 40.5%, respectively) and ${\widehat{\sigma }}_{s3}^{2}$ were 238.64, 66.19, and 159.85 (47.6, 33.0, and 71.2%, respectively) for TRL, SRL and DRL, respectively. The estimated row (${\widehat{\sigma }}_{r}^{2}$), spatial (${\widehat{\sigma }}_{s1}^{2}$, ${\widehat{\sigma }}_{s2}^{2}$, and ${\widehat{\sigma }}_{s2}^{2}$), and residuals (${\widehat{\sigma }}_{e}^{2}$) variances with RM2 were, in general, larger than for RM1. The three model effects, row (${\varvec{r}}$), spatial (${\varvec{s}}$), and residual (${\varvec{e}}$), described the environmental variance in RM1 and RM2.

An analysis of residuals was performed for root traits (Additional file 4: Figure S7). Normal distribution and homoscedasticity of residual variances were observed for all traits.

### Genetic parameters for root traits

The narrow (${\widehat{h}}^{2}$) and broad sense (${\widehat{H}}^{2}$) heritabilities at half-row level were estimated for RM1 and RM2 (Table 2). The ${\widehat{h}}^{2}$ and ${H}^{2}$ for root traits were low, and they were lower than for above-ground traits. The ${\widehat{h}}^{2}$ and ${H}^{2}$ had similar values within each trait due to the ${\widehat{\sigma }}_{l}^{2}$ being close to zero for all root traits, and all genetic variation was mostly explained by ${\widehat{\sigma }}_{g}^{2}$. The highest ${\widehat{h}}^{2}$ was observed for TRL, followed by SRL and the lowest ${\widehat{h}}^{2}$ was observed for DRL. In RM1, the ${\widehat{h}}^{2}$ for TRL was 0.046 (time-point 1 and 2) and 0.024 (time-point 3); for SRL it was 0.027 (time-point 1), 0.030 (time-point 2), and 0.025 (time-point 3); and for DRL it was 0.011 (time-point 1), 0.008 (time-point 2), and 0.010 (time-point 3). The RM2 presented a higher ${\widehat{h}}^{2}$ than RM1. In RM2, the ${\widehat{h}}^{2}$ for TRL was 0.046 (time-point 1 and 2) and 0.040 (time-point 3); for SRL was 0.28 (time-point 1 and 3) and 0.029 (time-point 3); and for DRL it was 0.012 (time-point 1), 0.011 (time-point 2), and 0.010 (time-point 3).

The genetic coefficient of variation ($GCV$) was estimated for RM1 and RM2 (Table 2), and similar values were observed for both models. The RM1 had a $GCV$ of 9.4, 10.2, and 6.5 for TRL, SRL, and DRL, respectively, and the RM2 had a $GCV$ of 8.8, 10.4, and 4.7 for TRL, SRL, and DRL, respectively. Generally, the $GCV$ observed for root traits were higher than for above-ground traits.

### Genomic predictions for above-ground traits

**Fig. 2:**
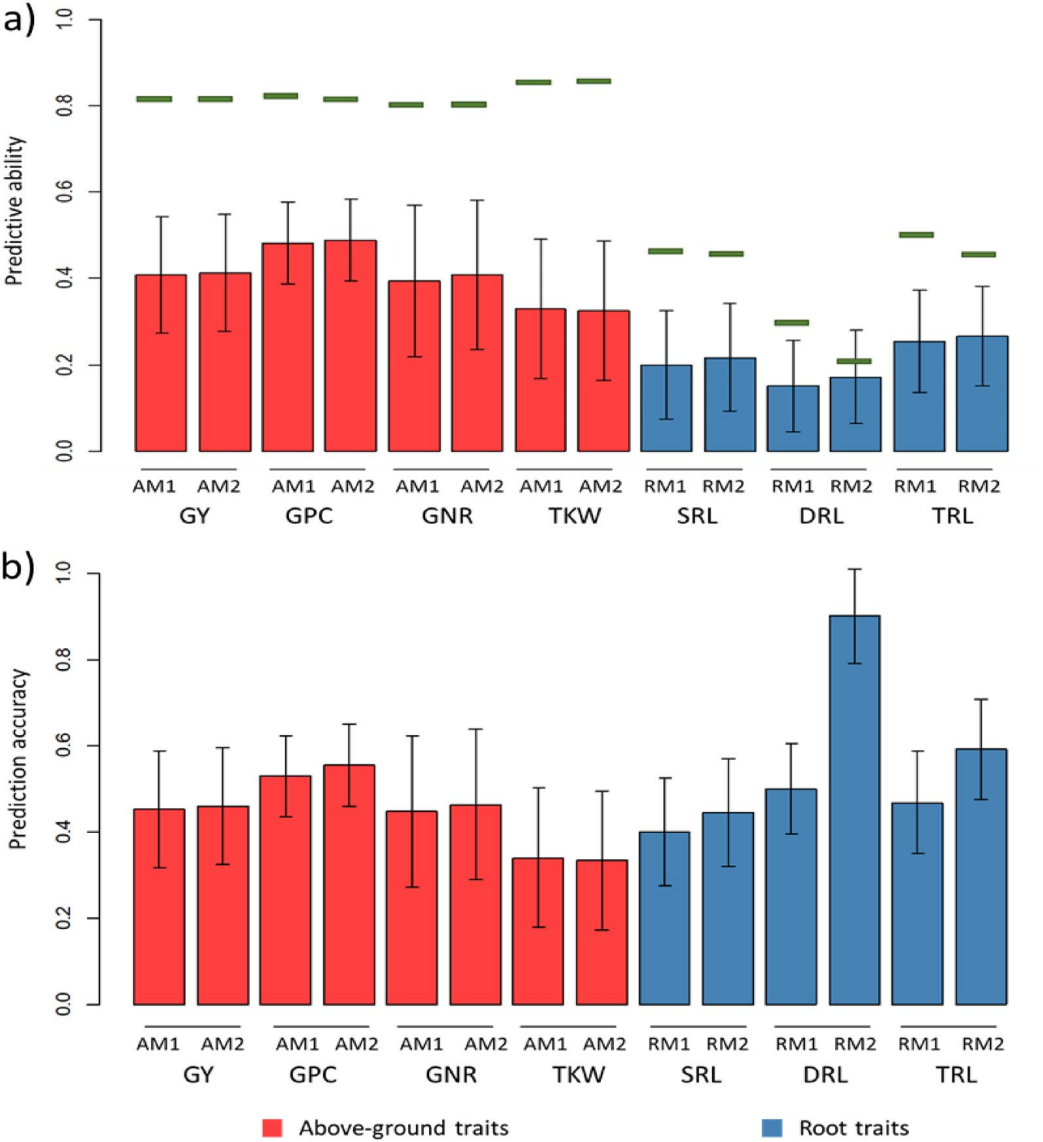
Barplot of predictive abilities for above-ground and root traits in leave-one-line out cross-validation. M1: spatial model 1 based on ${{\varvec{S}}}_{knn}$ correlation structure, M2: spatial model 2 based on ${{\varvec{S}}}_{euc}$ correlation structure, *GY* grain yield, *GPC* grain protein content, *GNR* grain nitrogen removal, *TKW* thousand kernel weight, *TRL* total root length, *SRL* shallow root length, *DRL* deep root length. Black bars represent the 95% confidence interval (CI) computed for each estimate (CI: standard deviation based on bootstrap sampling × 1.96). Green lines are the theoretical maximum PAs

**Fig. 3:**
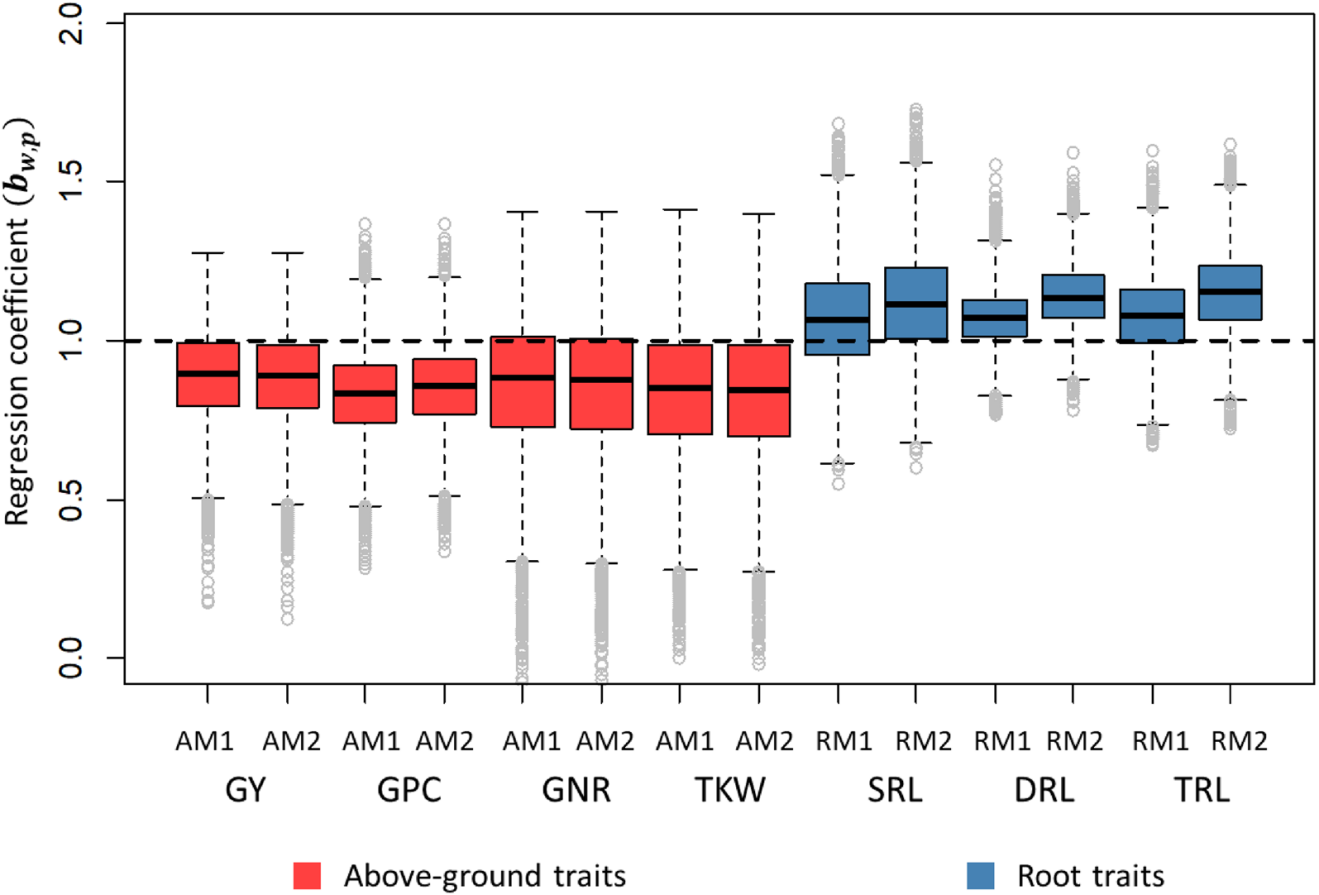
Boxplot of bootstrap distribution for the slope of the regression of additive genetic values obtained with whole phenotypic information on additive values obtained with partial phenotypic information (${{\varvec{b}}}_{w,p}$) in leave-one-line out cross-validation. M1: spatial model 1 based on ${{\varvec{S}}}_{knn}$ correlation structure, M2: spatial model 2 based on ${{\varvec{S}}}_{euc}$ correlation structure, *GY* grain yield, *GPC* grain protein content, *GNR* grain nitrogen removal, *TKW* thousand kernel weight, *TRL* total root length, *SRL* shallow root length, *DRL* deep root length. The black dashed line represents a regression coefficient of one, where no under or over-dispersion is present

The performance of genomic prediction (GP) was determined for AM1 and AM2 using LOO-CV. The predictive abilities (PAs) and prediction accuracy (ACC) for above-ground traits are presented in Fig. 3. The PAs for AM1 were 0.408, 0.482, 0.394, and 0.331 for GY, GPC, GNR and TKW, respectively, and for AM2 were 0.414, 0.489, 0.408, and 0.325 for GY, GPC, GNR and TKW, respectively. The AM2 had slightly higher PA than AM1 for GY, GPC and GNR. The differences in PA between M1 and M2 were significant in a Hotelling-Williams (significance threshold set at 0.01). The theoretical maximum PAs for AM1 and AM2 were similar between models for the different above-ground traits (green bars in Fig. 2). The ACC (Fig. 2b) followed a similar trend as the PA for all above-ground traits.

The regression coefficient of additive genetic values obtained with whole phenotypic information on additive values obtained with partial phenotypic information (${{\varvec{b}}}_{w,p}$) in LOO-CV is presented in Fig. 3. The ${{\varvec{b}}}_{w,p}$ is as an estimate for variance inflation in the predicted genetic effect, where ${{\varvec{b}}}_{w,p}$ = 1 reveals no under- or over-dispersion (i.e., no variance inflation for ${{\varvec{b}}}_{w,p}$ = 1). The bootstrap-based distribution of estimates revealed that ${{\varvec{b}}}_{w,p}$ was close to 1 for all above-ground traits with both models, indicating no significant variance inflation. Nevertheless, ${{\varvec{b}}}_{w,p}$ values around 0.9 for the different above-ground traits indicated a low over-dispersion (variance inflation) for predicted values.

### Genomic prediction for root traits

The performance of GP was evaluated for RM1 and RM2 using a LOO-CV. The PAs and ACC for root traits are presented in Fig. 2. The RM1 had a PA of 0.199, 0.153, and 0.250 for SRL, DRL and TRL, respectively, and the RM2 had a PA of 0.216, 0.171, and 0.267 for SRL, DRL, and TRL, respectively. The RM2 showed an improvement in PA compared to RM1 of 11.8, 8.9, and 6.5% for SRL, DRL and TRL, respectively, and differences between M1 and M2 were significant in a Hotelling-Williams (significance threshold set at 0.01). The theoretical maximum PAs for RM1 was higher than for RM2. Note that the theoretical maximum PAs depend on ${\widehat{h}}^{2}$, and therefore, the higher values observed for RM1 reflect the higher ${\widehat{h}}^{2}$ found for RM1. The ACC for root traits is presented in Fig. 2b. Higher ACCs were observed for RM2 compared to RM1, and larger differences between models were observed for ACC than for the PA.

The regression coefficients (${{\varvec{b}}}_{w,p}$) for RM1 and RM2 in LOO-CV are presented in Fig. 3. The bootstrap-based distribution of estimates revealed that ${{\varvec{b}}}_{w,p}$ was close to 1 for all root traits with both models, indicating that no significant variance inflation was present for any of the root traits. Nevertheless, the value around 1.1 for all root traits indicated a low under-dispersion (variance deflation) for predicted breeding values.

## Discussion

The present study was carried out in a semi-field (rain-out shelter) phenotyping facility (RadiMax) with four specific aims. First, we investigated the genetic variation and parameters for root development (shallow and deep) and four above-ground traits relevant to barley breeding (GY, GPC, GNR and TKW). Second, we studied the genotype-by-treatment (wet and dry) interaction for the root and above-ground traits in the RadiMax facility, and no relevant genotype-by-treatment interaction was detected for the different traits. Third, genomic prediction (GP) was investigated, and we observed that GP is feasible for all the analyzed traits. All the analyses were performed comparing two alternative nearest-neighbor adjustments (NNA) to model spatial variation in RadiMax.

### Genetic parameters and variance estimates for above-ground traits

The heritabilities (${\widehat{h}}^{2}$ and ${\widehat{H}}^{2}$) and variance components (VCs) were estimated for above-ground traits. In our study, ${\widehat{h}}^{2}$ and ${\widehat{H}}^{2}$ were reported for wet and dry conditions at the half-row level and not as family heritability, as sometimes are used in plant breeding [29]. Our estimates of heritabilities do not account for the number of repetitions used in the experiment. Therefore, the estimates are expected to be lower than family heritability, but they are more suitable to compare across studies and populations. The differences in ${\widehat{h}}^{2}$ and ${\widehat{H}}^{2}$ for wet and dry treatments were small and were attributed to heterogeneous spatial variance observed for each treatment, which resulted in a different phenotypic variance (${\widehat{\sigma }}_{P}^{2}$). The estimates of ${\widehat{h}}^{2}$ for GY ranged from 0.21 to 0.23, were in a similar range of previous estimates of 0.24 found by Tsai et al. [63] for a larger field population from Nordic Seed A/S breeding program, and by Ahmadi et al. [1]. The ${\widehat{h}}^{2}$ for GPC varied from 0.21 to 0.35, where the upper range values were obtained using AM2. The ${\widehat{h}}^{2}$ reported for GPC agreed with Nielsen et al. [41], who found a value of 0.21 using a Nordic Seed A/S breeding population. The ${\widehat{h}}^{2}$ for GNR in our population ranged from 0.16 to 0.21; other studies have reported family ${\widehat{h}}^{2}$ for GNR, as found in Schmidt et al. [55], but as previously discussed, the plot and family ${\widehat{h}}^{2}$ are not directly comparable. The TKW was the trait with the highest ${\widehat{h}}^{2}$ ranging from 0.50 to 0.54. Other studies have reported the family ${\widehat{h}}^{2}$ for TKW [4, 52].

The variance components for genotype-by-treatment interaction effects (${\varvec{t}}{\varvec{g}}$ and ${\varvec{t}}{\varvec{l}}$) were close to zero, meaning no relevant interaction effects were detected. Similar results have been found for wheat by Guo et al. [24] in a similar experiment in the RadiMax facility. The lack of interaction effects may be attributed to how the water stress gradient was managed in the experiment. According to a previous study using the same barley population in RadiMax, Svane et al. [59] observed that by the time the plants should have shown water stress symptoms, there was sufficient water available. Consequently, it delayed the start of water stress symptoms reducing the possibility of genotype-by-treatment interactions.

The genetic coefficient of variation ($GCV$) was estimated for all traits. The $GCV$ is a useful genetic parameter as it allows us to infer the potential of selection for the traits of interest and to compare the genetic variation across traits and populations. The $GCV$ were 6.9, 3.3, 6.8, and 5.7 for GY, GPC, GNR and TKW, respectively (average of AM1 and AM2), and no large differences were observed between AM1 and AM2. The same traits were investigated for a wheat breeding population in RadiMax facility [24], and the authors found $GCV$ estimates is an similar range for all traits.

### Genetic parameters and variance estimates for root traits

The heritabilities (${\widehat{h}}^{2}$ and ${\widehat{H}}^{2}$) and VCs were estimated for root traits in the three time points. The estimates of ${\widehat{h}}^{2}$ for TRL, SRL, and DRL were low and mainly dominated by the large row (${\widehat{\sigma }}_{r}^{2}$) and spatial (${\widehat{\sigma }}_{s1}^{2}$, ${\widehat{\sigma }}_{s2}^{2}$, and ${\widehat{\sigma }}_{s3}^{2}$) variation observed. The ${\widehat{h}}^{2}$ ranged from 0.02 to 0.04 for TRL, 0.02 to 0.03 for SRL, and 0.002 to 0.01 for DRL. Comparing between time points, higher ${h}^{2}$ was seen for time-point 1, which was measured 36 days before the last time-point. The differences in the ${h}^{2}$ between time points were explained by the heterogeneous spatial variance, resulting in a different ${\widehat{\sigma }}_{P}^{2}$ for each time point. Other studies have reported ${\widehat{h}}^{2}$ for root traits [31, 51], but they have focused on family mean ${\widehat{h}}^{2}$ instead of half-row ${\widehat{h}}^{2}$ as in our study.

The $GCV$ was estimated for TRL, SRL, and DRL. In our study, the $GCV$ is a particularly relevant parameter for root traits as it allows us to interpret the genetic variation independently from the environmental variables affecting the population. The $GCV$ were 9.1, 10.3, and 5.6 for TRL, SRL, and DRL, respectively (average of RM1 and RM2), and no large differences were observed for RM1 and RM2. The $GCV$ for TRL and SRL were larger than the above-ground traits analyzed. High values of $GCV$ are desirable for breeding as it means that there is good potential for selection and there will be a good response to selection for the analyzed traits. The $GCV$ for DRL was lower than the rest of the root traits, revealing a lower potential for breeding and response to selection.

### Genomic prediction for above-ground traits

Genomic prediction for AM1 and AM2 was evaluated for the above-ground traits using a LOO-CV (Fig. 2). The LOO-CV uses the largest possible training population, which maximize the genetic correlations between training and testing sets. The LOO-CV allows us to compare and investigate the potential PA of genetic models. The PA, measured as the correlation between corrected phenotypes $({y}_{c})$ and GEBVs, was used as an estimate of the correlation between GEBV and true underlying breeding values. The AM1 and AM2 showed a similar PA and ACC for the above-ground traits, with a trend of slightly higher PA and ACC for AM2 to GY, GPC and GNR. The PAs observed in our study were in the range reported in previous studies for GY [18] and GPC [41, 55], and were higher for GY, PC, and GNR than PAs reported for a five-fold CV in Hansen et al. [25]. For TKW, we observed similar PA than Hansen et al. [25] and lower PA than Schmid and Thorwarth [54] and Thorwarth et al. [60], which found PAs around 0.7. The higher PA obtained for TKW in other studies could be related to performing GP on populations coming from a single breeding program. Note that genetic relations between lines are higher within breeding programs (e.g. due to full and half-sibs are included in the population) than between breeding programs. Consequently, higher genetic relationships are present between individuals in TP and VP, resulting in higher PA. The differences in PA among traits could be attributed to several factors, among them, the LD (linkage disequilibrium) between markers in training and testing populations, the heritability of the trait under investigation, and the genetic architecture of traits, where complex traits controlled by many loci with small effects have lower predictability than traits controlled with less number of loci [26, 53]. Another relevant parameter in GS is the regression coefficients ${{\varvec{b}}}_{w,p}$, which is used as an estimate for variance inflation in the predicted genetic effect. In general no large variance inflation was observed as ${{\varvec{b}}}_{w,p}$ was not statistically different from 1 for all above-ground traits (Fig. 3; nevertheless, a low over-dispersion (inflation in ${{\varvec{b}}}_{w,p}$ was reported (${{\varvec{b}}}_{w,p}$ ~0.9). The variance infaltion could be attributed to having a mixture of different breeding populations (and breeding cycles) in the experiment, which could result in differences in allele frequency and LD for individuals genetically distant.

### Genomic prediction for root traits

Genomic prediction for RM1 and RM2 was evaluated for root traits using a LOO-CV (Fig. 2). The PAs for root traits were lower than for above-ground traits (Fig. 2a). The highest PA was observed for TRL, followed by SRL, and the lowest was for DRL. Higher PA for RM2 was observed, and differences between models were significant in a Hotelling-Williams test (significance threshold set at 0.01). The improvement in PA conferred by RM2 was 11.8, 8.9, and 6.5% for SRL, DRL and TRL, respectively. Similarly, higher ACC were obtained with RM2. The differences between RM1 and RM2 were acentuated for the ACC estimate (Fig. 2b). This can be explained due to the estimate of ACC is inversely related to ${\widehat{h}}^{2}$, and the lower ${\widehat{h}}^{2}$ values found fot RM2, contributed to higher ACC obtained with RM2. Our results confirm that the accuracy of predicting genetic effects for root development in barley is sufficient to allow genetic selection. Other reports have demonstrated the viability of GP for root traits in barley [51] and other species [24, 35, 70].

An additional analysis was performed using a multi-trait model to study the genetic correlation ($\rho$) between SRL and DRL (results not shown) and revealed a positive correlation between the traits ($\rho =0.36$). The positive correlation is convenient for breeding since the better performance of GP for SRL could be exploited by selecting higher values on SRL, leading to higher DRL.

The regression coefficients (${{\varvec{b}}}_{w,p}$) were used to estimate variance inflation in the predicted genetic effect for root traits (Fig. 3). In general, ${{\varvec{b}}}_{w,p}$ was close to 1, revealing no relevant variance inflation. Nevertheless the ${{\varvec{b}}}_{w,p}$ ~1.1 indicated a low under-dispersion (deflation) for predicted values, which could be explained by having a mixture of different breeding populations in the experiment, resulting in differences in allele frequency and LD for individuals genetically distant.

### Modeling of spatial effects in genetic models

Two spatial methodologies (M1 and M2) based on NNA [47, 68] using 5-left and -right neighbors and (co)variance structures (${{\varvec{S}}}_{knn}$ or ${{\varvec{S}}}_{euc}$) to model spatial variation were utilized. The M1 and M2, differ in the distance function used to compute the correlation between neighbors (Additional file 3: Figure S4). The ${{\varvec{S}}}_{knn}$ connected the row of the observation with the 5-left and -right neighbors for each observation; while the ${{\varvec{S}}}_{euc}$ is a more developed adjustment which starts connecting the target rows to the 5-left and -right neighbors, followed by weighing neighbors’ relationships according to the Euclidean distance between them and the target row. In general, both methodologies (either for above-ground or root traits) presented similar trends for the different traits. In some cases, VCs were similar between the methodologies, but differences in allocating variance were observed in some specific cases. Other studies, as Guo et al. [24] for barley and Malinowska et al. [35] for perennial ryegrass modeled spatial variation in RadiMax, with methodologies comparable to M1. Similarly to our work, they have concluded that the spatial variation was significant and accounting for spatial effects was needed to reduce the noise level. In addition to the results presented, an analysis without including virtual rows was performed. A relevant influence of virtual rows was observed in VCs analysis, decreasing the variance captured by spatial effects when they were not included. The CVs analysis revealed better predictive performance for M2 (especially for root traits), and it is therefore, our preferred methodology for the RadiMax experiment.

## Conclusions

In this research, we utilized data from a semi-field phenotyping facility (RadiMax) to investigate above-ground and root traits of spring barley under a water availability gradient. First, we concluded that heritable genetic variation and significant genetic coefficient of variation ($GCV$) were present, indicating a good breeding potential for all analyzed traits. Second, no relevant genotype-by-treatment (wet and dry) interaction and genetic effects due to neighboring lines were observed in RadiMax. Third, we concluded that there is good potential to perform genomic prediction for all analyzed traits, as revealed by the high predictive ability and prediction accuracy, and low variance inflation of predicted genetic effect in the leave-one-line-out cross-validation analysis. Fourth, all the performed analyses were carried out using two proposed spatial methodologies, and the main conclusion was that our most developed spatial methodology had a significant effect on predictive performance, improving genomic prediction especially for root traits.

### Supplementary Information

**Additional file 1: Figure S1.** Principal component analysis (PCA) of genomic relationship lines. **Figure S2.** Heatmap of genomic relationship matrix.

**Additional file 2: Figure S3.** Boxplot of above-ground and root traits after data edition. *GY* grain yield, *GPC* grain protein content, *GNC* grain nitrogen content, *TKW* thousand kernel weight, *SRL* shallow root length, *DRL* deep root length, *TRL* total root length

**Additional file 3: Figure S4.** Scatter plot of the between-neighbor correlations in M1 (${{\varvec{S}}}_{knn}$) and M2 (${{\varvec{S}}}_{euc}$) as a function of plot distances. **Figure S5.** Heatmap for the spatial correlation structure ${{\varvec{S}}}_{euc}$. Example for time-point 1 and subbed 1.

**Additional file 4: Figure S6.** Histogram and scatter plots for residuals of above-ground traits (example for AM1). *GY* grain yield, *GPC* grain protein content, *GNC* grain nitrogen content, *TKW* thousand kernel weight. **Figure S7.** Histogram and scatter plots for residuals of root traits (example for RM1). TRL: total root length; SRL: shallow root length; DRL: deep root length.

**Additional file 5: Table S1.** Standard errors of variance estimates for above-ground traits. **Table S2.** Standard errors of variance estimates for root traits.

## Data Availability

The datasets analyzed in the current study are available at the Harvard dataverse public repository at the following link for phenotyipic (https://doi.org/10.7910/DVN/KZZSRH) and genomic information (https://doi.org/10.7910/DVN/W1QIJR).

## References

[1] Ahmadi J, Vaezi B, Pour-Aboughadareh A (2016). Analysis of variability, heritability, and interrelationships among grain yield and related characters in barley advanced lines. Genetika.

[2] Baenziger PS, Depauw RM (2009). Wheat breeding: Procedures and strategies Wheat science and trade.

[3] Bhatta M, Gutierrez L, Cammarota L, Cardozo F, Germán S, Gómez-Guerrero B (2020). Multi-trait genomic prediction model increased the predictive ability for agronomic and malting quality traits in barley (*Hordeum*
*vulgare* L). Genes Genomes Genetics.

[4] Bouhlal O, Affricot JR, Puglisi D, El-Baouchi A, El Otmani F, Kandil M (2022). Malting quality of ICARDA elite winter barley (*Hordeum*
*vulgare* l) germplasm grown in Moroccan middle atlas. J Am Soc Brewing Chem.

[5] Buerstmayr H, Ban T, Anderson JA (2009). QTL mapping and marker-assisted selection for Fusarium head blight resistance in wheat: a review. Plant Breeding.

[6] Burgueño, J. (2018). Spatial analysis of field experiments. Applied statistics in agricultural biological and environmental sciences. Madison.

[7] Collard BC, Mackill DJ (2008). Marker-assisted selection: an approach for precision plant breeding in the twenty-first century. Phil Trans Royal Soc B Biol Sci.

[8] Costa Silva J, Potts B, Gilmour A, Kerr R (2017). Genetic-based interactions among tree neighbors: identification of the most influential neighbors, and estimation of correlations among direct and indirect genetic effects for leaf disease and growth in Eucalyptus globulus. Heredity.

[9] Crossa J, Campos Gde L, Perez P, Gianola D, Burgueno J, Araus JL (2010). Prediction of genetic values of quantitative traits in plant breeding using pedigree and molecular markers. Genetics.

[10] Crossa J, Pérez-Rodríguez P, Cuevas J, Montesinos-López O, Jarquín D, De Los Campos G (2017). Genomic selection in plant breeding: methods, models, and perspectives. Trends Plant Sci.

[11] Cullis B, Gleeson A (1991). Spatial analysis of field experiments-an extension to two dimensions. Biometrics.

[12] Cullis B, Gogel B, Verbyla A, Thompson R (1998). Spatial analysis of multi-environment early generation variety trials. Biometrics.

[13] Cuyabano BCD, Rovere G, Lim D, Kim TH, Lee HK, Lee SH (2021). GPS coordinates for modelling correlated herd effects in genomic prediction models applied to hanwoo beef cattle. Animals.

[14] Den Herder G, Van Isterdael G, Beeckman T, De Smet I (2010). The roots of a new green revolution. Trends Plant Sci.

[15] Dray S, Blanchet G, Borcard D, Guenard G, Jombart T, Larocque G (2018). Package ‘adespatial’. R package.

[16] Dreisigacker S, Sukumaran S, Guzmán C, He X, Lan C, Bonnett D (2016). Molecular marker-based selection tools in spring bread wheat improvement: CIMMYT experience and prospects. Molecular Breeding for Sustainable Crop Improvement:.

[17] Dunn OJ, Clark V (1971). Comparison of tests of the equality of dependent correlation coefficients. J Am Stat Assoc.

[18] Endelman JB, Atlin GN, Beyene Y, Semagn K, Zhang X, Sorrells ME (2014). Optimal design of preliminary yield trials with genome-wide markers. Crop Sci.

[19] Fè, D., Ashraf, B.H., Pedersen, M.G., Janss, L., Byrne, S., Roulund, N., et al. (2016). Accuracy of genomic prediction in a commercial perennial ryegrass breeding program. *The Plant Genome* 9(3)**,** plantgenome2015.2011.0110. 10.3835/plantgenome2015.11.0110 27902790

[20] Francia E, Tacconi G, Crosatti C, Barabaschi D, Bulgarelli D, Aglio E (2005). Marker assisted selection in crop plants. Plant Cell Tissue Organ Cult.

[21] Gilmour AR, Cullis BR, Verbyla AP (1997). Accounting for natural and extraneous variation in the analysis of field experiments. J Agric Biol Environ Stat.

[22] Gleeson AC, Cullis BR (1987). Residual maximum likelihood (REML) estimation of a neighbour model for field experiments. Biometrics.

[23] Goddard M, Hayes B (2007). Genomic selection. J Anim Breed Genet.

[24] Guo X, Svane SF, Füchtbauer WS, Andersen JR, Jensen J, Thorup-Kristensen K (2020). Genomic prediction of yield and root development in wheat under changing water availability. Plant Methods.

[25] Hansen PB, Ruud AK, de Los Campos G, Malinowska M, Nagy I, Svane SF (2022). Integration of DNA methylation and transcriptome data improves complex trait prediction in hordeum vulgare. Plants.

[26] Hayes BJ, Pryce J, Chamberlain AJ, Bowman PJ, Goddard ME (2010). Genetic architecture of complex traits and accuracy of genomic prediction: coat colour, milk-fat percentage, and type in Holstein cattle as contrasting model traits. PLoS Genet.

[27] Hertel TW, Burke MB, Lobell DB (2010). The poverty implications of climate-induced crop yield changes by 2030. Glob Environ Chang.

[28] Hinkelmann K, Kempthorne O (2007). Design and analysis of experiments Introduction to experimental design.

[29] Holland, J.B., Nyquist, W.E., Cervantes-Martínez, C.T., and Janick, J. (2003). Estimating and interpreting heritability for plant breeding: an update. Plant breeding reviews 22.

[30] ISO-16634 (2016). Food products—Determination of the total nitrogen content by combustion according to the Dumas principle and calculation of the crude protein content. International Organization for Standardization.

[31] Jia Z, Liu Y, Gruber BD, Neumann K, Kilian B, Graner A (2019). Genetic dissection of root system architectural traits in spring barley. Front Plant Sci.

[32] Legarra A, Reverter A (2018). Semi-parametric estimates of population accuracy and bias of predictions of breeding values and future phenotypes using the LR method. Genet Sel Evol.

[33] Lobell DB, Schlenker W, Costa-Roberts J (2011). Climate trends and global crop production since 1980. Science.

[34] Madsen, P., and Jensen, J. (2013). "An User's Guide to DMU, Version 6, Release 5.1. Center for Quantitative Genetics and Genomics," in Dept. of Molecular Biology and Genetics, University of Aarhus. Research Centre Foulum Tjele, Denmark).

[35] Malinowska M, Ruud AK, Jensen J, Svane SF, Smith AG, Bellucci A (2022). Relative importance of genotype, gene expression, and DNA methylation on complex traits in perennial ryegrass. Plant Genome.

[36] Marjanovic J, Mulder HA, Rönnegård L, Bijma P (2018). Modelling the co-evolution of indirect genetic effects and inherited variability. Heredity.

[37] Marjanovic J, Mulder HA, Rönnegård L, de Koning DJ, Bijma P (2022). Capturing indirect genetic effects on phenotypic variability: competition meets canalization. Evol Appl.

[38] Martin R (1996). 15 Spatial experimental design. Handbook Statist.

[39] Meuwissen THE, Hayes BJ, Goddard ME (2001). Prediction of total genetic value using genome-wide dense marker maps. Genetics.

[40] Miedaner T, Korzun V (2012). Marker-assisted selection for disease resistance in wheat and barley breeding. Phytopathology.

[41] Nielsen NH, Jahoor A, Jensen JD, Orabi J, Cericola F, Edriss V (2016). Genomic prediction of seed quality traits using advanced barley breeding lines. PLoS ONE.

[42] Olesen JE, Trnka M, Kersebaum KC, Skjelvåg AO, Seguin B, Peltonen-Sainio P (2011). Impacts and adaptation of European crop production systems to climate change. Eur J Agron.

[43] Papadakis, J. (1937). Méthode statistique pour des expériences sur champ. Thessalonike: Institut d'Amélioration des Plantes à Salonique.

[44] Pham A-T, Maurer A, Pillen K, Brien C, Dowling K, Berger B (2019). Genome-wide association of barley plant growth under drought stress using a nested association mapping population. BMC Plant Biol.

[45] Philipp N, Liu G, Zhao Y, He S, Spiller M, Stiewe G (2016). Genomic prediction of barley hybrid performance. Plant Genome.

[46] Piepho HP, Boer MP, Williams ER (2022). Two-dimensional P-spline smoothing for spatial analysis of plant breeding trials. Biom J.

[47] Piepho HP, Richter C, Williams E (2008). Nearest neighbour adjustment and linear variance models in plant breeding trials. Biom J.

[48] Raffo M, Azzimonti G, Pereyra S, Pritsch C, Lado B, Dreisigacker S (2022). Introgression of the coupled Fhb1-Sr2 to increase Fusarium head blight and stem rust resistance of elite wheat cultivars. Plant Genetic Resources.

[49] Raffo M, Sarup P, Andersen J, Orabi J, Jahoor A, Jensen J (2022). Integrating a growth degree-days based reaction norm methodology and multi-trait modeling for genomic prediction in wheat. Front n Plant Sci.

[50] Ribaut J-M, Hoisington D (1998). Marker-assisted selection: new tools and strategies. Trends Plant Sci.

[51] Robinson H, Kelly A, Fox G, Franckowiak J, Borrell A, Hickey L (2018). Root architectural traits and yield: exploring the relationship in barley breeding trials. Euphytica.

[52] Rode J, Ahlemeyer J, Friedt W, Ordon F (2012). Identification of marker-trait associations in the German winter barley breeding gene pool (Hordeum vulgare L). Mol Breeding.

[53] Sallam A, Endelman J, Jannink JL, Smith K (2015). Assessing genomic selection prediction accuracy in a dynamic barley breeding population. Plant Genome.

[54] Schmid KJ, Thorwarth P (2014). Genomic selection in barley breeding. Biotechnol Approaches Barley Improve.

[55] Schmidt M, Kollers S, Maasberg-Prelle A, Großer J, Schinkel B, Tomerius A (2016). Prediction of malting quality traits in barley based on genome-wide marker data to assess the potential of genomic selection. Theor Appl Genet.

[56] Smith A, Cullis B, Thompson R (2001). Analyzing variety by environment data using multiplicative mixed models and adjustments for spatial field trend. Biometrics.

[57] Stringer JK, Cullis BR, Thompson R (2011). Joint modeling of spatial variability and within-row interplot competition to increase the efficiency of plant improvement. J Agric Biol Environ Stat.

[58] Svane SF, Dam EB, Carstensen JM, Thorup-Kristensen K (2019). A multispectral camera system for automated minirhizotron image analysis. Plant Soil.

[59] Svane SF, Jensen CS, Thorup-Kristensen K (2019). Construction of a large-scale semi-field facility to study genotypic differences in deep root growth and resources acquisition. Plant Methods.

[60] Thorwarth P, Ahlemeyer J, Bochard A-M, Krumnacker K, Blümel H, Laubach E (2017). Genomic prediction ability for yield-related traits in German winter barley elite material. Theor Appl Genet.

[61] Townley-Smith T, Hurd E (1973). Use of moving means in wheat yield trials. Can J Plant Sci.

[62] Tsai H-Y, Cericola F, Edriss V, Andersen JR, Orabi J, Jensen JD (2020). Use of multiple traits genomic prediction, genotype by environment interactions and spatial effect to improve prediction accuracy in yield data. PLoS ONE.

[63] Tsai H-Y, Janss LL, Andersen JR, Orabi J, Jensen JD, Jahoor A (2020). Genomic prediction and GWAS of yield, quality and disease-related traits in spring barley and winter wheat. Sci Rep.

[64] VanRaden PM (2008). Efficient methods to compute genomic predictions. J Dairy Sci.

[65] Velazco JG, Rodríguez-Álvarez MX, Boer MP, Jordan DR, Eilers PH, Malosetti M (2017). Modelling spatial trends in sorghum breeding field trials using a two-dimensional P-spline mixed model. Theor Appl Genet.

[66] Verbyla AP, Cullis BR, Kenward MG, Welham SJ (1999). The analysis of designed experiments and longitudinal data by using smoothing splines. J Roy Stat Soc: Ser C.

[67] Verbyla AP, De Faveri J, Wilkie JD, Lewis T (2018). Tensor cubic smoothing splines in designed experiments requiring residual modelling. J Agric Biol Environ Stat.

[68] Wilkinson G, Eckert S, Hancock T, Mayo O (1983). Nearest neighbour (NN) analysis of field experiments. J Roy Stat Soc: Ser B.

[69] Wood TB, Stratton F (1910). The interpretation of experimental results. J Agric Sci.

[70] Yonis BO, Pino del Carpio D, Wolfe M, Jannink J-L, Kulakow P, Rabbi I (2020). Improving root characterisation for genomic prediction in cassava. Sci Rep.

