# Supplementary material for: Genomic prediction for root and yield traits of barley under a water availability gradient: a case study comparing different spatial adjustments
Source: Plant Methods. 2024 Jan 12;20:8. doi: 10.1186/s13007-023-01121-y (PMC10785381; doi:10.1186/s13007-023-01121-y)
Supplement: Supplementary file 1 — Additional file 1: Figure S1. Principal component analysis (PCA) of genomic relationship lines. Figure S2. Heatmap of genomic relationship matrix. [file 13007_2023_1121_MOESM1_ESM.docx]

**Supplementary material 1**

In this material, we present a Principal Component Analysis (PCA) for genotypes based on genomic information (Figure S1), and a Heatmap of the genomic relationship matrix (Figure S2). In both figures, a common trend of lines in two sub-groups can be observed, corresponding to more family-related lines within breeding companies. In addition, some isolated lines not clustered in any of the sub-groups are observed.


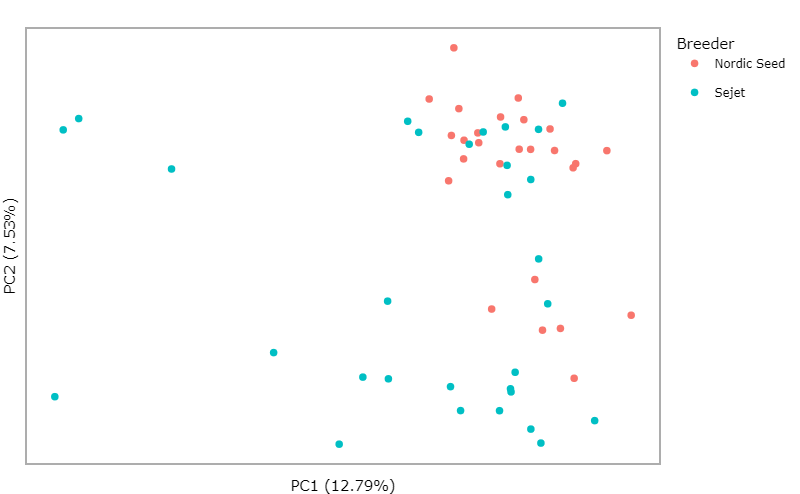


**Figure S1.** Principal component analysis (PCA) of genomic relationship lines.


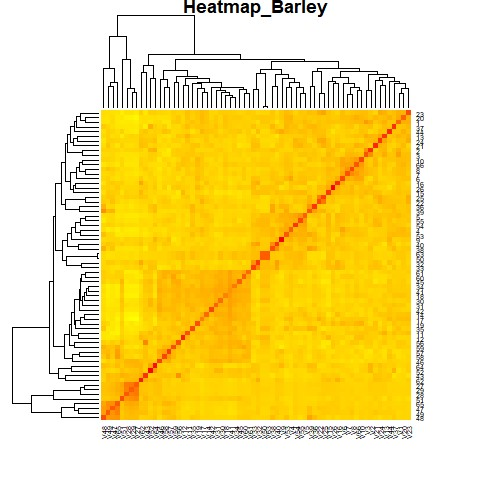


**Figure S2.** Heatmap of genomic relationship matrix.
