# Supplementary material for: Genomic prediction for root and yield traits of barley under a water availability gradient: a case study comparing different spatial adjustments
Source: Plant Methods. 2024 Jan 12;20:8. doi: 10.1186/s13007-023-01121-y (PMC10785381; doi:10.1186/s13007-023-01121-y)
Supplement: Supplementary file 2 — Additional file 2: Figure S3. Boxplot of above-ground and root traits after data edition. GY grain yield, GPC grain protein content, GNC grain nitrogen content, TKW thousand kernel weight, SRL shallow root length, DRL deep root length, TRL total root length [file 13007_2023_1121_MOESM2_ESM.docx]

**Supplementary material 2**


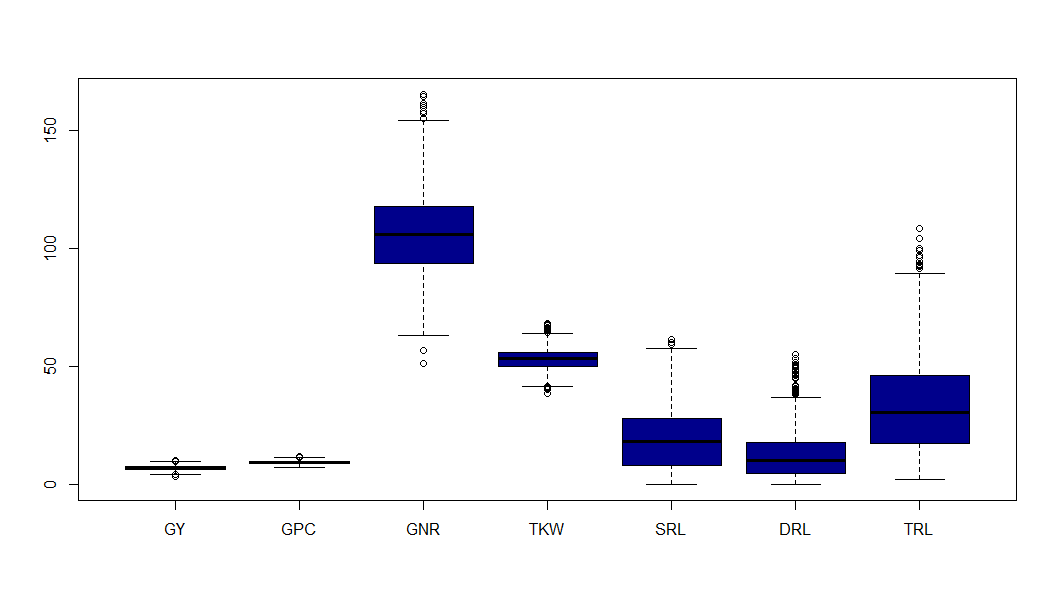


**Figure S3**. Boxplot of above-ground and root traits after data edition. GY: grain yield, GPC: grain protein content, GNC: grain nitrogen content, TKW: thousand kernel weight, SRL: shallow root length, DRL: deep root length, TRL: total root length.
