# Supplementary material for: Genomic prediction for root and yield traits of barley under a water availability gradient: a case study comparing different spatial adjustments
Source: Plant Methods. 2024 Jan 12;20:8. doi: 10.1186/s13007-023-01121-y (PMC10785381; doi:10.1186/s13007-023-01121-y)
Supplement: Supplementary file 3 — Additional file 3: Figure S4. Scatter plot of the between-neighbor correlations in M1 (${{\varvec{S}}}_{knn}$Sknn) and M2 (${{\varvec{S}}}_{euc}$Seuc) as a function of plot distances. Figure S5. Heatmap for the spatial correlation structure ${{\varvec{S}}}_{euc}$Seuc. Example for time-point 1 and subbed 1. [file 13007_2023_1121_MOESM3_ESM.docx]

**Supplementary material 3**

In this material, we present a scatter plot of the between-neighbor correlations in $\boldsymbol{S}_{knn}$ (M1) and $\boldsymbol{S}_{euc}$ (M2) as a function of neigbors distance (Figure S4), The “0” in the x-axis represent the row of the observation. The correlation in both models goes until the neighbor 10; after that, correlations decrease to zero. The blue and red curves in the plot can be seen as the distance function behind the between-neighbor correlations for M1 and M2, respectively, The $\boldsymbol{S}_{euc}$ correlation structure weight higher correlations for closer neighbors and lower for more distant neighbors compared to $\boldsymbol{S}_{knn}$

**
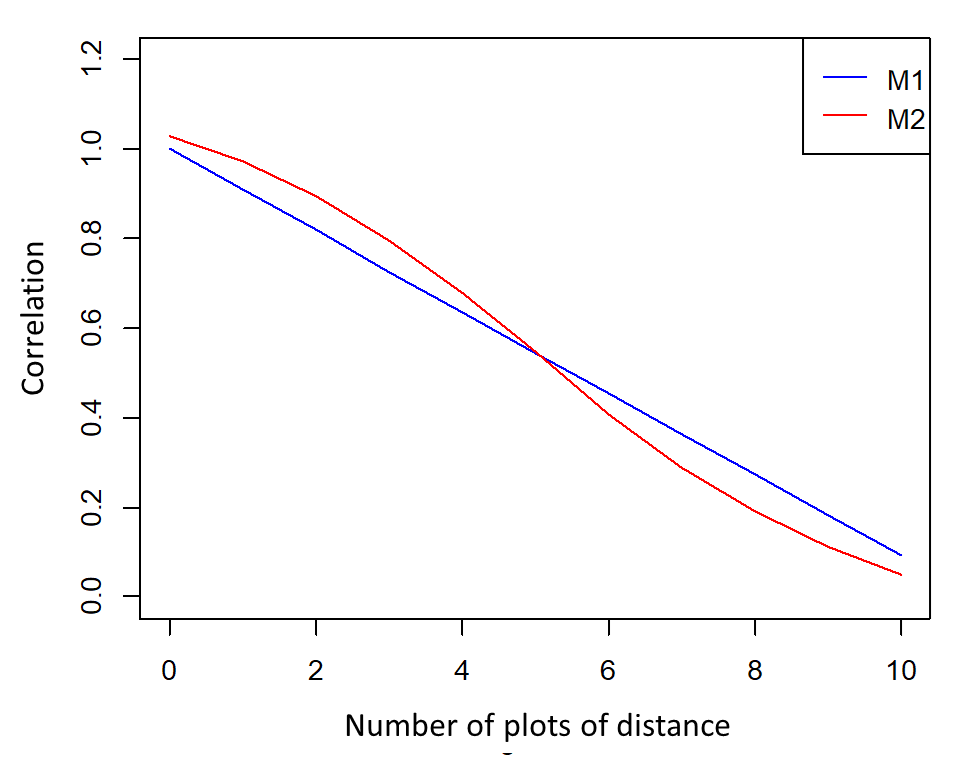
Figure S4**. Scatter plot of the between-neighbor correlations in M1 ($\boldsymbol{S}_{knn}$) and M2 ($\boldsymbol{S}_{euc}$) as a function of plot distances.

The Figure S5 shows an example of a heatmat of $\boldsymbol{S}_{euc}$ for time-point 1 and subbed 1.

**Figure S5**. Heatmap for the spatial correlation structure $\boldsymbol{S}_{euc}$. Example for time-point 1 and subbed 1.
