# Supplementary material for: Genomic prediction for root and yield traits of barley under a water availability gradient: a case study comparing different spatial adjustments
Source: Plant Methods. 2024 Jan 12;20:8. doi: 10.1186/s13007-023-01121-y (PMC10785381; doi:10.1186/s13007-023-01121-y)
Supplement: Supplementary file 4 — Additional file 4: Figure S6. Histogram and scatter plots for residuals of above-ground traits (example for AM1). GY grain yield, GPC grain protein content, GNC grain nitrogen content, TKW thousand kernel weight. Figure S7. Histogram and scatter plots for residuals of root traits (example for RM1). TRL: total root length; SRL: shallow root length; DRL: deep root length. [file 13007_2023_1121_MOESM4_ESM.docx]

**Supplementary material 4**

**Figure S6**. Histogram and scatter plots for residuals of above-ground traits (example for AM1). GY: grain yield, GPC: grain protein content, GNC: grain nitrogen content, TKW: thousand kernel weight.

**Figure S7**. Histogram and scatter plots for residuals of root traits (example for RM1). TRL: total root length; SRL: shallow root length; DRL: deep root length.
